# Smart Microneedles for Therapy and Diagnosis

**DOI:** 10.34133/2020/7462915

**Published:** 2020-12-18

**Authors:** Xiaoxuan Zhang, Yuetong Wang, Junjie Chi, Yuanjin Zhao

**Affiliations:** ^1^State Key Laboratory of Bioelectronics, School of Biological Science and Medical Engineering, Southeast University, Nanjing 210096, China; ^2^Department of Rheumatology and Immunology, Institute of Translational Medicine, The Affiliated Drum Tower Hospital of Nanjing University Medical School, Nanjing 210008, China

## Abstract

Microneedles represent a cutting-edge and idea-inspiring technology in biomedical engineering, which have attracted increasing attention of scientific researchers and medical staffs. Over the past decades, numerous great achievements have been made. The fabrication process of microneedles has been simplified and becomes more precise, easy-to-operate, and reusable. Besides, microneedles with various features have been developed and the microneedle materials have greatly expanded. In recent years, efforts have been focused on generating smart microneedles by endowing them with intriguing functions such as adhesion ability, responsiveness, and controllable drug release. Such improvements enable the microneedles to take an important step in practical applications including household drug delivery devices, wearable biosensors, biomedical assays, cell culture, and microfluidic chip analysis. In this review, the fabrication strategies, distinctive properties, and typical applications of the smart microneedles are discussed. Recent accomplishments, remaining challenges, and future prospects are also presented.

## 1. Introduction

Microneedles are a type of miniaturized medical device which contain an array of microsized needles with heights no more than 1 mm and sharp tips [1, 2]. As they could penetrate the epidermal layer, generate channels through the skin, and avoid the contact with blood capillaries and nerves in the meanwhile, microneedles are minimally invasive, almost painless, and anti-infectious [3, 4]. Although microneedles are less suitable for precisely targeting at specific sites when compared with other minimally invasive technologies such as laser and ultrasound scalpel, they are more mild and less harmful to the human body [5]. Besides, microneedles are easier and more convenient for medical staff or even patients to operate. Additionally, it is feasible to combine microneedles with other diagnosis/treatment systems and potential to employ microneedles in a wide range of applications [3, 6]. Benefitting from these advantages, microneedles have attracted increasing interest in scientific researches and medical fields, including real-time biomarker detection and transdermal drug delivery [7]. By employing flexible template-based molding, top-down processing methods like etching, and bottom-up strategies like 3D printing [8], a large variety of microneedles such as hollow microneedles, solid microneedles, and multilayer microneedles from diverse materials including metal, glass, and hydrogel have been fabricated and put into applications [6]. Today, microneedles could not only efficiently deliver proteins, small molecules, liquid drugs, and other drugs to multiple parts of the human body for disease treatment but also associate with paper chips, microfluidic systems, and other techniques for sensitive detection. Besides, microneedles could also carry cells and facilitate cell culture, cytology tests, cytokine delivery, and so on [9, 10].

In recent years, to further improve their performances and make them qualified for the high, strict requirements of real-time applications, much effort has been devoted to developing new-generation smart microneedles. These smart microneedles are bionic, bioderived, or biocompatible and have unique properties (e.g., skin adhesion, dissolvability, responsiveness, and tip-substrate detachability) [11, 12] for meeting the demands in different application scenarios (e.g., wearable devices, rapid delivery, responsive delivery or detection, and sustained delivery). These microneedles could possess ideal adhesion ability and good conformability to the skin and are capable to be applied to flexible, highly active, or frequently motional body parts. Also, these microneedles are promising to break the bottlenecks of low delivery efficiency, unavoidable drug waste, and simple delivery modes. Additionally, due to the improvements of the microneedle materials, the biosafety of these microneedles could be guaranteed, making them an ideal medical device for medical use. Thus, such microneedles are believed to play a significant part in a wide range of biomedical fields, inspiring the combination of microneedles and other practical devices such as household medical products, portable real-time detectors, and wearable biosensors [13]. In the future, more efforts will be devoted to the development of innovative biomaterial-composed microneedles with new properties (e.g., multiresponsiveness, theragnostic integration, and stepwise drug delivery) and broad applications (e.g., cell culture, wound healing, microfluidics, and flexible electronics). Promoting the translation of microneedles from laboratory to market is also an important goal for the future.

In this paper, we provide an overview focusing on the advances of smart microneedles with distinctive features, their fabrication methods, outstanding functions, and practical applications. After briefly introducing the general fabrication strategies, we present detailed descriptions about the novel properties and designs of these microneedles. Their advanced applications in biomedical engineering are then discussed. Examples of representative microneedles are also listed, and their strengths are emphasized in this review. Finally, we give a conclusion and views on the current limitations and future direction of the development of microneedles.

## 2. Advanced Fabrication Strategies

After being conceptualized in the 1950s [14], microneedles have experienced rapid development, especially in the past 20 years. Since then, various microneedles have been produced, which can be classified into metal microneedles, glass microneedles, silicon microneedles, ceramic microneedles, and polymer microneedles based on the materials [6, 12]. Due to the large choices of materials, high processability, and ideal biocompatibility, the new-generation smart microneedles are normally polymer microneedles. With the aim of generating polymer microneedles with desirable functions and demand-meeting features on a large scale, a vast number of advanced fabrication strategies have been proposed [8, 9]. Mold-based fabrication, as a commonly used, reusable, and highly accurate method nowadays, is not only making possible the generation of complicatedly structured microneedles such as multilayer microneedles and tip-substrate separable microneedles but also realizing the massive production [15]. Another emerging strategy is mold-free fabrication like drawing lithography and droplet-born air blowing, which gets rid of the restrictions of molds and thus is regarded as versatile, flexible, scalable, and economical [16–18]. A summary of the advantages and limitations of each strategy as well as their potential in different applications is given in Table 1.

### 2.1. Mold-Based Fabrication

**Figure 1:**
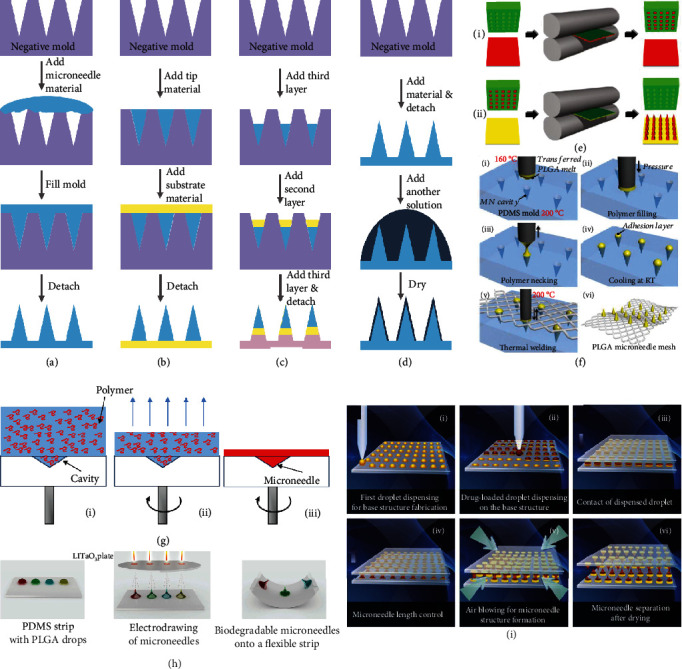
Schematic illustrations of fabrication strategies. (a) A typical four-step fabrication method. (b) Fabrication of two-layer microneedles. (c) Fabrication of three-layer microneedles. (d) Fabrication of coating microneedles. (e) Two-step heating transfer molding: (i) mold filling and tip solidification; (ii) tip-substrate connection. Reproduced with permission from Ref. [24], copyright 2013, Wiley-VCH. (f) Transfer molding to fabricate microneedles on a surgical mesh. Reproduced with permission from Ref. [25], copyright 2017, Elsevier B.V. (g) Spin coating process. Reproduced with permission from Ref. [26], copyright 2017, Elsevier B.V. (h) Electrodrawing method. Reproduced with permission from Ref. [17], copyright 2014, Wiley-VCH. (i) Droplet-born air blowing method. Reproduced with permission from Ref. [18], copyright 2013, Elsevier B.V.

A typical mold-based fabrication contains four steps (Figure 1(a)) [15]. The first step is to obtain a master structure with the same structure as the microneedles by using approaches such as etching, laser ablation, 3D printing, and micromachining. Then in the second step, a negative mold is made by casting curable materials (e.g., polydimethylsiloxane, PDMS) over the master structure and peeling them off after they are solidified. During this process, the features of the master structure can be well retained by the negative mold. The third step is to fill prepolymer or other microneedle materials into the negative mold by means of vacuuming, centrifuging, and so on. For the final step, the microneedles are polymerized, detached from the negative mold, and preserved. Up to now, such mold-based fabrication technology has been further modified and improved so that more types of microneedles can be produced based on this method [19].

For example, by filling different materials into the negative mold step by step, microneedles with heterogeneous properties can be easily fabricated (Figure 1(b) and (c)). Gu et al. provided a two-layer microneedle patch for diabetes treatment, whose 600 *μ*m high glucose-responsive tips were strong enough to penetrate the skin, while the soft substrate was unresponsive and acted as a supporting layer [20]. Also, Park et al. designed a three-layer microneedle patch with a total height of about 600 *μ*m [21]. The water-soluble substrate was chosen as the first layer, allowing the implantation of tips into the skin. The second layer was the drug-containing water-soluble polymer for rapid drug release, and the third layer was composed of drug-loading biodegradable polymer for sustained drug delivery. In addition, metal, silicon, and glass can also be the substrate [22]. With metal shafts as the substrate, the polymer solution as the connection, and the drug solution as the tips, arrowhead-shaped microneedles were presented for sustainable, long-term drug release.

The prepared microneedles can be further modified through surface coating [3, 23]. To be specific, a solution containing molecules, proteins, DNA, or other bioactive substances is added to the microneedle surface and gets dried (Figure 1(d)). The most distinctive advantage of these coated microneedles is that they can avoid the destruction of bioactive substances during the microneedle fabrication process and thus ensure the bioactivity. Besides, coating is one of the simplest and most controllable approaches to make microneedles functional. Particularly, for microneedles with detection abilities, it facilitates the sampling, isolation, and detection of specific biomarkers to coat biological probes on the surface.

For the aforementioned microneedles, vacuum treatment and centrifugation were employed to fill microneedle materials into the negative mold. In addition to these filling methods, other means such as imprinting, spin coating, infiltrating, and spraying have also been used [24–27]. A typical example of imprinting is to use a heated nip to transfer tip materials [24]. For instance, polyvinylpyrrolidone (PVP) microneedles presented by DeSimone's group were fabricated by a two-step transference. To get PVP tips, a PVP film-negative mold mate was first passed through a heated nip at 105°C to allow for the mold filling and tip solidification. Then to connect the substrate and the tips and to detach the microneedles from the mold, a hydrosoluble substrate together with the filled mold was passed through the heated nip again at 65°C (Figure 1(e)). The resultant microneedles were measured 361.4 ± 5.7 *μ*m in height. Also, when replacing the heated nip with a heated micropillar, the tip material can be pressed into the cavities of the negative mold and transferred to the substrate in turns, as shown in Figure 1(f) [25].

The spin coating method is based on gravity action, liquid flow, and solvent evaporation [26]. Generally, the material solution will fill the cavities because of gravity during the spin coating process (Figure 1(g)). In the meanwhile, a quantity of solution flows off the mold and the liquid film becomes thinner and thinner, which then accelerates the solvent evaporation and enormously decelerates the liquid flow. Thus, the liquid flow gradually ceases, the evaporation continues, and polymer molecule deposits in the cavities as well as the mold surface until the formation of microneedles. On the other hand, infiltrating takes advantage of the concentration gradient to attract polymer molecules to the cavities. Besides, spraying makes use of fine droplets to overcome the high interfacial tension between the solution and the mold. Combining these two methods, Vrdoljak et al. produced 500 *μ*m high dissolvable microneedles containing trivalent inactivated influenza vaccine for immunization [27]. During the fabrication process, deionized water was first sprayed to the negative mold to fill the cavities. After the removal of excess water, a mixture of polymer molecules and a highly concentrated vaccine was then applied directly on top of the cavities. Owing to the concentration gradient, the mixture would diffuse in the cavities and reach a balance. Then by evaporating the solvent and employing an adhesive substrate to detach the tips, the final microneedle product was obtained.

### 2.2. Mold-Free Fabrication

Although lots of progress and improvements have been made, the mold-based fabrication strategy still faces some restrictions. The multiple steps involved in mold-based fabrication are costly, time-consuming, and technology-dependent. Besides, during the mold-filling procedure, drug loss remains a challenge. Also, as the shape and size of each negative mold are fixed and unchangeable, it usually requires different negative molds to fabricate different microneedles, which is inflexible, nonuniversal, and inconvenient. To overcome these limitations, mold-free fabrication of microneedles has been proposed. Also, numerous approaches have been put forward, including drawing lithography [16, 17], droplet-born air blowing [18], centrifugal lithography [28], 3D printing [29], and microlens-integrated technique [30].

The drawing lithography technique takes advantage of the stretching deformation of a viscous polymer in its glassy transition, which is an intermediate state between the solid state and the liquid state [16]. During this process, the polymer (e.g., maltose) is initially melted at a high temperature. Then after the temperature gradually decreases below the melting point of the polymer, the drawing will begin and stop at specific temperature points with specific speed. Sometimes, such a drawing procedure will be repeated several times. These drawn microstructures will finally be completely solidified when the temperature cooled down to the glass transition temperature of the polymer, and desired microneedles with heights more than 400 *μ*m will be fabricated. For further improvement, a noncontacting, room-temperature electrodrawing has been proposed using an electrohydrodynamic process [17]. There are three key steps for the electrodrawing method, which are droplet deposition, electrohydrodynamic force-driven drawing, and solvent evaporation (Figure 1(h)). Specifically, polymer droplets are first deposited orderly on a substrate. A driving plate with the pyroelectric effect is then placed above the droplets and produces an appropriate electric field to arouse electrohydrodynamic force. Induced by such force, the droplets deform into the conical microneedle shape and this shape will be retained during the evaporation of the solvent, thus forming the microneedles. Notably, with the droplet volume between 0.05 *μ*L and 0.1 *μ*L, the microneedle height would be between 300 *μ*m and 500 *μ*m; when the droplet volume ranges from 0.3 *μ*L to 1.8 *μ*L, the microneedle height would range from 400 *μ*m to 800 *μ*m. The superiority of the drawing lithography approach is that it overcomes the limitations of traditional mold-based methods without multiple repeated processes, harmful steps, and drug loss. Besides, this approach also possesses many other advantages including easy engineerization, high fabrication efficiency, flexible sizes and shapes, and potential for massive production.

Different from drawing lithography, droplet-born air blowing depends on the direct application of air blowing to polymer droplets for solidification and microneedle formation [18]. For a typical procedure, the base polymer droplets and the tip polymer droplets are deposited on flat plates successively (Figure 1(i)). Two of these plates are put face to face, and the upper one moves downward to contact the bottom one. After contacting, the upper plate then moves upward and is kept at a predetermined distance from the bottom plate, which decides the length of the microneedles. To dry the polymer droplets and shape the microneedle structure, air blowing is then applied. Finally, the completely solidified microneedles will separate from each other and two microneedle patches can be fabricated at one time. In one example, the height of the prepared microneedles was 600 *μ*m. Additionally, another polymer solidification strategy is to employ a suitable centrifugal force to evaporate the solvent [28]. Particularly, the polymer droplets are dispensed on a micropillar array, which not only facilitates the centrifugation but also improves the skin penetration behavior and drug delivery efficiency of the microneedles.

3D printing is a promising and versatile approach for elaborate synthesis and has been widely used for fabricating complicated patterns, microstructures, and functional materials [29, 30]. The introduction of 3D printing into manufacturing microneedle-related systems has aroused extensive attention, and much effort has been devoted. For example, researchers presented a 3D-printed 1000 *μ*m high microneedle array composed of photosensitive class I resin, which was approved by the FDA [29]. It was also demonstrated that such microneedles could successively deliver insulin to the skin. In addition, it is found that by employing microlens to direct the direction of light and focus light into a conical path, microneedles can be mass-produced in an inexpensive and mold-free way [30]. Notably, the height, diameter, and shape of the microneedles can be precisely controlled and flexibly adjusted by choosing appropriate parameters such as the diameter and the curvature of the microlens. For instance, conical microneedles with 750 *μ*m in height and 150 *μ*m in diameter have been fabricated with this method [30].

## 3. Unique Properties and Designs

The most basic properties of microneedles are breaking through the skin barrier, enhancing skin permeability, and contacting with skin interstitial fluid in a noninvasive, painless way [9]. These, however, can no longer meet the demands of controllable drug release, highly efficient skin interstitial fluid extraction, versatile application on diverse organs, long-term wearability, convenient operation processes, etc. Thus, it is significant to design novel microneedles and impart them with advanced and desirable properties. Up to now, many kinds of smart microneedles have been designed and fabricated, such as adhesive microneedles [31], rapidly dissolvable microneedles [2], separable microneedles [11, 22], and responsive microneedles [20, 32]. These properties can make the smart microneedle potential as household medical devices for detection and therapy. For instance, the adhesion ability helps the microneedles tightly fix to the applied parts and become wearable to constantly monitor physiological indices or deliver drugs. Besides, dissolvable microneedles, separable microneedles, and responsive microneedles can meet the practical demands of rapid release of drugs in a large quantity, sustained and moderate drug release, and intelligent and controllable drug release, respectively.

### 3.1. Adhesion Ability

Due to the tension and repulsion of skin, general microneedles cannot tightly adhere to the skin without external forces or additional medical supplies, whereas the endowment of skin adhesion ability to microneedles enables them to overcome such restrictions and helps them stick to the skin during application, which greatly benefits consistent long-term drug delivery or biosensing [31]. Besides, the body parts where adhesive microneedles are applied can move as usual, improving the comfort of users. Typically, there are three ideas to prepare adhesive microneedles: designing specially structured microneedle tips [33], choosing swellable materials to fabricate microneedle tips [34, 35], and making the supporting layer adhesive [36].

#### 3.1.1. Specially Structured Microneedle Tips

**Figure 2:**
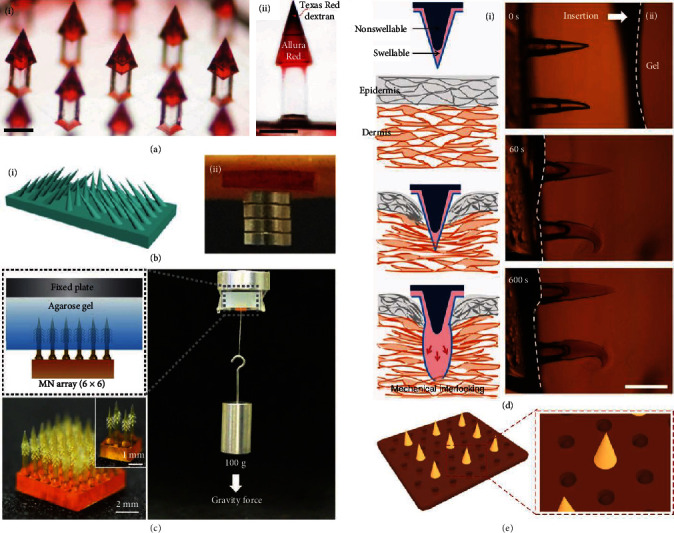
Adhesive microneedles. (a) Optical images of arrow-shaped microneedles. Both scale bars are 250 *μ*m. Reproduced with permission from Ref. [33], copyright 2020, Elsevier B.V. (b) Serrated clamping microneedles: (i) schematic illustration of the clamping microneedles; (ii) digital photo of microneedles that bears 4 small magnet blocks adhering to porcine skin. Reproduced with permission from Ref. [19], copyright 2019, Science China Press. (c) Optical image of the barbed microneedle array and the adhesion force tests. Reproduced with permission from Ref. [31], copyright 2020, Wiley-VCH. (d) Schematic illustrations (i) and optical images (ii) of mechanical interlocking of swellable microneedles after penetration. The scale bar is 500 *μ*m. Reproduced with permission from Ref. [35], copyright 2013, Springer Nature. (e) Schematic illustrations of microneedles with polydopamine hydrogel supporting layer and suction-cup-like microstructures. Reproduced with permission from Ref. [36], copyright 2020, American Association for the Advancement of Science.

To ensure successful skin adhesion and retention, arrow-shaped microneedles have been presented, as shown in Figure 2(a) [33]. When applied to the skin, the pyramidal structure of microneedle tips inhibited the withdrawal of microneedles, thus making microneedles adhere to and retain in the skin. As the tips gradually dissolved in the skin, drugs or other cargos carried by microneedles were released sustainably and continuously. In addition, researchers get inspirations from nature and introduce natural structures to adhesive microneedles [19, 31]. For example, Zhao et al. mimicked the serrated microstructure of mantises' forelegs and fabricated a serrated clamping microneedle array (Figure 2(b)). Strikingly, all the tips of such microneedles slanted toward the central row and formed an angled clamping structure. This structure greatly improved the skin adhesion ability, and the microneedle array was demonstrated to bear the weight of more than 4 small magnet blocks. Another example was the barbed microneedle array, which imitated feeding ducts of mosquitoes, proboscises of endoparasitic worms, stingers of honeybees, and quills of porcupines (Figure 2(c)). The backward-facing barbs on the microneedle tips could interlock with the tissue and hold a weight of 100 g without detaching from the attached tissue model.

#### 3.1.2. Swellable Microneedle Materials

Typically, swellable microneedles comprise tips derived from swellable materials, which react upon contact with skin interstitial fluid and cause localized tissue interlocking [34]. For instance, Yang and coworkers developed microneedles with swellable tips (poly(styrene)-block-poly(acrylic acid)) and nonswellable cores (polystyrene), as shown in Figure 2(d) [35]. Via covalent bonds, the swellable tips and the nonswellable cores could strongly connect with each other. By water-led volume expansion, the microneedles achieved mechanical interlocking with tissues and universal soft tissue adhesion. It was found that the adhesion forces of these microneedles were about three times higher than their counterparts.

#### 3.1.3. Adhesive Supporting Layer

In addition to designing the features of microneedle tips, developing microneedle supporting layers with special materials or structures can also generate adhesive microneedles [36]. In terms of materials, biocompatible adhesive hydrogels such as chitosan gel, tannin gel, and polydopamine gel are ideal candidates for microneedle supporting layers. As for structures, bioinspired microsuction cups, hexagonal projections, etc., have been proved to extensively improve the adhesion ability of polymer patches. For example, Zhao's group chose polydopamine copolymer as the microneedle supporting layer and further integrated octopus-mimicked suction-cup-shaped chambers with their microneedle patches (Figure 2(e)). The resultant patches showed ideal adhesion capacity in dry, moist, wet, and underwater environments, thus potential to be applied to various situations of daily usage.

### 3.2. Dissolvability

There are various advantages to employ rapidly dissolvable materials as the microneedle tips [37–45]. Firstly, drugs would release along with the dissolution of microneedle tips and the total dissolving time is normally less than 30 min. These attributes make the dissolvable microneedles attain the purpose of delivering drugs rapidly and massively, especially for vaccine delivery. Besides, the drugs loaded in microneedles can be almost completely released, thus avoiding the drug waste caused by the incomplete release. In addition, benefitting from their dissolvability, these microneedles do not leave remains of fractured microneedle tips in the skin, minimizing the risk of microneedle breakage. Moreover, infection probability can be also decreased as the penetrated skin return nearly to normal before the removal of dissolvable microneedles. In general, the dissolvable materials for microneedle tips include PVP [2], HA [37], PVA [38], CMC [43], trehalose [44], and *γ*-PGA [45], most of which originate from organisms and are water-soluble, nontoxic, nonirritating, biocompatible, and tractable. A summary of the representative tip materials, how they are fabricated, and how long they dissolve is given in Table 2.

### 3.3. Separability

Once applied to the skin, the supporting layer of separable microneedles will detach from the tips and be removed, leaving only the tips inside the skin [46–48]. This can substantially shorten the wear time of microneedles and prevent the possible inconvenience and irritation resulted from long-term wearing. Notably, these separable microneedles are especially suitable for slow sustained drug release when it requires to remain sufficient drug concentration for a long period. Two strategies have been employed to generate separable microneedles. One is to break off the tips from the supporting layer by weakening their connection and applying external forces [11, 46], while the other is to dissolve or degrade the supporting layer [1, 47].

#### 3.3.1. Fracture of Tip and Supporting Layer

By using different kinds of weakly sticky materials as the microneedle tips and the supporting layer to weaken their connection, the fabricated separable microneedles can achieve immediate tip fracture during insertion and retraction. For example, a microneedle array has been reported to have HA as its tip material and biocompatible polycaprolactone (PCL) as its supporting layer material, respectively [46]. Due to the weak adhesion between HA and PCL, almost all tips were left inside the skin after the removal of the supporting layer. In spite of the high separation rate and facile operation, such design brings lots of trouble to the microneedle fabrication, as it is difficult to completely detach these microneedles from the negative mold.

**Figure 3:**
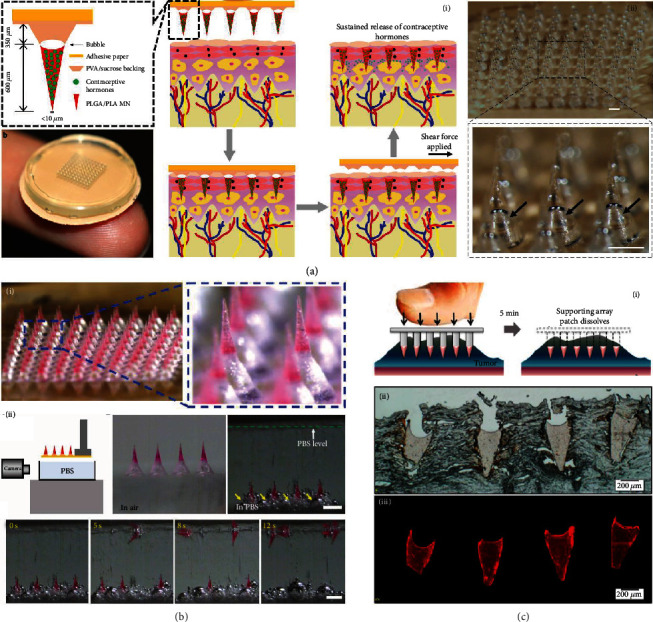
Separable microneedles. (a) Separable microneedles with bubble structures: (i) schematic illustrations of the composition and application of the microneedles; (ii) optical images of bubble-containing microneedles. The scale bars are 500 *μ*m. Reproduced with permission from Ref. [11], copyright 2019, Springer Nature. (b) Effervescent microneedles with separable ability: (i) optical images of the microneedles; (ii) the separation process of effervescent microneedles in PBS solution. The scale bars are 500 *μ*m. Reproduced with permission from Ref. [48], copyright 2019, American Association for the Advancement of Science. (c) Separable microneedles with dissolvable supporting layer: (i) schematic illustrations of the separation process; (ii, iii) histological sections of microneedle tips left inside the tissue. Reproduced with permission from Ref. [32], copyright 2016, American Chemical Society.

To solve this problem, Li et al. designed a bubble structure between each microneedle tip and the supporting layer [11]. The microneedle tips that contained 20% *w*/*w* levonorgestrel (a long-acting contraceptive hormone) were first solidified, and an air bubble was entrapped within each microneedle tip after drying of the aqueous supporting layer solution. The resultant microneedles were demonstrated to efficiently penetrate the skin under the vertical force and break off when the shear force was applied (Figure 3(a)). Notably, the separation rate was more than 95% and the left microneedle tips could sustainably deliver levonorgestrel with relatively constant release kinetics of about 0.3%-2.2% per day *in vitro*. The same group further introduced an effervescent formulation, which mainly consisted of sodium bicarbonate and citric acid, in the supporting layer (Figure 3(b)) [48]. Upon contacting with skin interstitial fluid, sodium bicarbonate and citric acid would instantly react and produce CO_2_ bubbles to destroy the attachment of microneedle tips to the supporting layer, leading to the tip fracture. Results showed that it took less than 10 s to separate the tips from the supporting layer in the PBS solution and less than 1 min within the skin. The *in vivo* levonorgestrel pharmacokinetics were also assessed. The levonorgestrel in rat plasma was found to reach the peak within 3 days, stay above the human therapeutic threshold level for over 30 days, and decrease to zero after 60 days. The most distinctive feature of this design is that the separation is derived from harmless, benign, and autonomous chemical reactions without the involvement of mechanical effects, thus contributing to more convenient usage.

#### 3.3.2. Dissolvable Supporting Layer

Another type of separable microneedles included a quickly dissolving supporting layer. Once the microneedles are inserted into the skin, the skin interstitial fluid rapidly dissolves the supporting layer and the microneedle tips are separated and embedded in the skin tissue. Taking advantage of such design, Ling et al. provided a light-activatable microneedle system for locoregional thermal therapy and controllable drug release (Figure 3(c)) [32]. The supporting layer was made from a dissolvable PVA/PVP mixture, while the microneedle tips were from heat responsive polycaprolactone (PCL) containing drugs and photothermal nanoparticles. After separation from the supporting layer, the embedded microneedle tips could generate heat in response to NIR irradiation, raising the temperature in the targeted tissue for therapy and melting PCL for broad drug release. The release profile of an anticancer drug doxorubicin under intermittent NIR irradiation was also investigated. A stepwise release profile was found, with a steep increase occurring at the laser-on state and almost no release appearing at the laser-off state. Besides, after all the cycles, the final release rate could be more than 90%.

### 3.4. Responsiveness

Responsive microneedles generate responses to external or internal stimulations; thus, they can realize a precise control of release of drugs or other microcargos. Besides, the release condition can also be adjusted based on varied physiological environments or recommended dosage, avoiding the side effects brought about by excessive release or efficacy loss resulting from inadequate release. Light is considered as a common external stimulus, and internal stimuli involve pH, specific biochemical molecules, etc.

#### 3.4.1. External Stimulus

By loading photosensitive nanomaterials such as lanthanum hexaboride, Prussian blue nanoparticles, and gold nanorod inside microneedle tips, microneedles can be endowed with near-infrared light (NIR) or ultraviolet light (UV) responsiveness [32, 49]. For instance, Chen et al. embedded silica-coated lanthanum hexaboride (LaB_6_@SiO_2_) inside microneedles for NIR-triggered transdermal drug delivery [50]. The local temperature around the microneedles was found to rise to 49°C within 10 s and 58°C within 30 s when NIR was applied. Owing to the high temperature, the microneedles quickly melted, leading to drug delivery. Despite the responsive, controllable drug release ability, the heat provided by the microneedles might be intolerable by human tissues. Thus, finding materials that could melt at a milder temperature and controlling the NIR dose would be the next research target. In addition, Hardy et al. encapsulated drugs in a light-responsive 3,5-dimethoxybenzoin conjugate and put them together inside microneedles [51]. These microneedles could act as “on-demand” delivery devices, which delivered drugs only under UV exposure and stopped releasing after the removal of UV light. This feature made them potential for use in situations like patient- or physician-controlled analgesia.

#### 3.4.2. Internal Stimulus

The last decade has witnessed the emergence and development of pH-responsive microneedles. Recently, a microneedle array composed of pH-sensitive cellulose acetate phthalate has been presented [52]. It was reported that cellulose acetate phthalate could stay stable in acid environments but would swell or even dissolve upon touching alkaline environments. Thus, microneedles consisting of such polymer swelled in response to the increase in local pH, leading to the delivery of the entrapped drugs. Based on these microneedles, researchers further built up an electrochemically controlled drug release platform by imposing a suitable reducing potential to provide a desired pH environment for drug release. This platform was demonstrated to be efficient and reliable and was anticipated to offer alternative means of dosage control.

**Figure 4:**
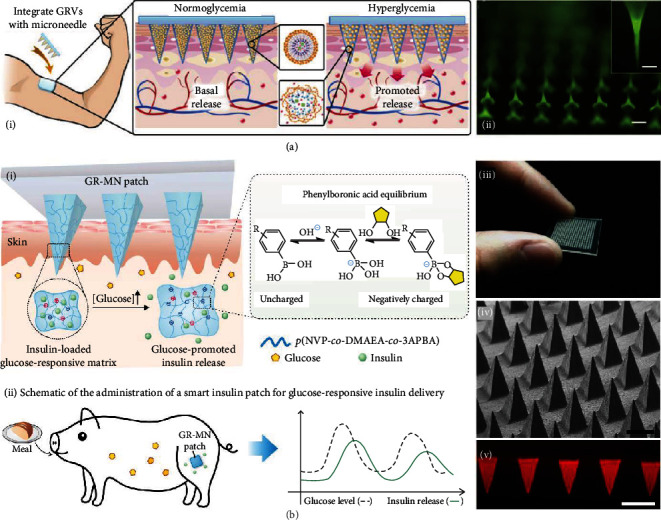
Glucose-responsive microneedle patch for insulin delivery. (a) Microneedles loaded with hypoxia-sensitive vesicles: (i) schematic illustrations of responsive insulin release from the microneedles; (ii) fluorescence microscopy image of microneedles carrying vesicles. Insulin is labelled with FITC. Both scale bars are 200 *μ*m. Reproduced with permission from Ref. [20], copyright 2015, PNAS. (b) Microneedles with a glucose-responsive matrix: (i) mechanisms of responsive insulin release from the microneedles; (ii) the application and effects of the microneedles on a diabetic pig; (iii) digital photo of the microneedle patch; (iv) SEM image; (v) fluorescence microscopy image. The scale bars are 500 *μ*m. Reproduced with permission from Ref. [53], copyright 2020, Springer Nature.

Microneedles can also be designed to intelligently release drugs or other cargos according to the presence or concentration of specific biochemical molecules to maintain the homeostasis of the human body. For years, researchers have been focusing on the development of glucose-responsive microneedles for insulin delivery. In 2015, the group of Yu presented a “smart insulin patch,” which was the first prototype of the closed-loop transdermal microneedle patch [20]. Such a patch was loaded with hypoxia-sensitive vesicles (Figure 4(a)). Glucose was oxidized by enzymes under the hyperglycemic environment, leading to a local hypoxic state, the dissolution of vesicles, and the release of loaded insulin. Recently, the same group further improved their smart insulin patch and applied it to minipigs [53]. During the application, excessive blood glucose reacted with phenylboronic acid units within the polymeric matrix of the microneedles to form negatively charged glucose-boronate complexes (Figure 4(b)). The increased negative charge not only contributed to the swelling of the polymeric matrix but also weakened the electrostatic attraction between insulin and the polymeric matrix, which brought about rapid insulin release. The release profiles of both insulin patches in diabetic mice showed that after the application of microneedles, the plasma insulin concentrations quickly rose to over 1000 *μ*U/mL, then gradually descended, and finally returned to normal after 12 h.

In addition to glucose, microneedles can also respond to other molecules including insulin, thrombin, and reactive oxygen species (ROS). One example of insulin-responsive microneedles was realized by a competitive mechanism [54]. Specifically, insulin and glucagon were connected via an insulin aptamer to form a conjugate. Such conjugate was immobilized on the HA matrix of microneedles through the interaction between insulin and HA. In the presence of a high insulin concentration, the ambient insulin would compete with the conjugate for the binding sites of the HA matrix, leading to a rapid glucagon release and efficiently preventing insulin-caused hypoglycemia. The *in vitro* release profile showed that glucagon would be released rapidly when incubated with high concentrations of insulin and that glucagon release was neglectable without insulin. When the insulin concentration was 1 mg/mL, about 125 *μ*g/mL glucagon was released in 1 h. Besides, thrombin-responsive microneedles were designed to deliver heparin when thrombin was activated [55]. These microneedles were demonstrated to achieve a successful feedback-controlled autoanticoagulant regulation. Additionally, ROS-responsive microneedles were fabricated for treating acne [56]. The overgenerated ROS within acne would degrade the tip material of the microneedles, and the loaded drugs would responsively and controllably release.

### 3.5. Antibacteria

Microneedles could be imparted with antibacterial ability and applied to treating bacterial infections. For this purpose, many kinds of microneedles have been prepared, which are composed of antibacterial materials like chitosan or contain antibacterial particles like silver nanoparticles, antibacterial peptides, and bioactive extracts [36]. For example, microneedles containing antimicrobial agents, chloramphenicol, were fabricated and employed to treat bacterial biofilms [57]. The microneedles could penetrate the biofilm, facilitating the chloramphenicol delivery, while the loaded chloramphenicol was released only in response to the activity of active bacterial community, significantly reducing the off-target toxicity. Such antibacterial microneedles were well applicable to treating wounds and other sites that were susceptible to infection.

### 3.6. Others

**Figure 5:**
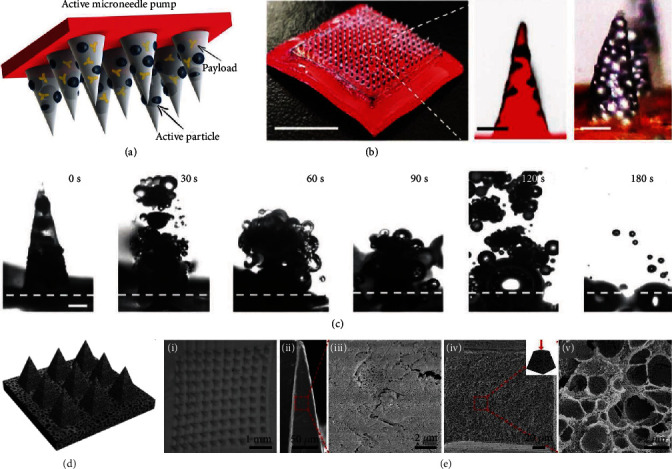
Explosive and porous microneedles. (a) Schematic illustration of the composition of active microneedles. (b) Digital photo of an active microneedle patch and optical/fluorescence microscopy images of an active microneedle tip. The scale bars are 300 *μ*m and 25 *μ*m, respectively. (c) Real-time images of the explosion of a single active microneedle tip in PBS solution. The scale bar is 200 *μ*m. Reproduced with permission from Ref. [58], copyright 2020, Wiley-VCH. (d) Schematic illustration of porous polymer microneedles. (e) SEM images of a porous microneedle array (i), the surfaces (ii, iii), and the cross-sections (iv, v) of a porous microneedle tip. Reproduced with permission from Ref. [59], copyright 2020, Royal Society of Chemistry.

For deeper, broader, and faster transdermal delivery, Lopez-Ramirez et al. designed and fabricated active microneedles, which exploded like volcanos upon contact with skin tissue [58]. Such explosive behavior of the active microneedles was attributed to the reaction between embedded magnesium particles and the skin interstitial fluid (Figure 5(a) and (b)). During the reaction, a large amount of H_2_ bubbles was rapidly produced, breaking up the microneedles and enhancing the release of the cargos (Figure 5(c)). Compared to the delayed delivery by passive microneedles without magnesium particles, the active microneedles exploded and dissolved within 2 min, greatly enhancing cargo delivery by both lateral and vertical routes. This “explosive” property made the active microneedles extremely suitable for deep and fast delivery, such as pain relief and hemostasis.

For more efficient skin interstitial fluid extraction and cargo loading, polymer microneedles with highly porous and interconnected microstructures have been generated. To fabricate porous microneedles, various approaches have been adopted, such as adding pore-forming agents to the prepolymers [60, 61] and changing the structure of the polymer chain [62]. In a recent study, Kim et al. used a “water annealing” method to modulate the polymorphism of their silk fibroin microneedles [63]. These microneedles were treated with water annealing for 4-8 h, and thus, their water solubility was decreased and fibrillar porous structures were increased. In addition, Liu et al. employed a “phase inversion” strategy to produce such porous polymer microneedles (Figure 5(d)) [59]. The scanning electron microscope (SEM) images of the surface and cross-section of the resultant microneedles clearly showed their porous and interconnected structures (Figure 5(e)). In comparison to normal microneedles, the porous microneedles were demonstrated to improve the skin interstitial fluid extraction efficiency by more than 5 times. Besides, as for the cargo-loading efficiency, it was found that porous microneedles with porosity of 45.8% could carry 43.2% of liquid solution [59]. Therefore, with the improved extraction and loading ability originated from the porous structure, these porous microneedles were potential to play a role in metabolic analysis, biomarker diagnosis, transdermal drug delivery, and other biomedical applications.

## 4. Biomarker Detection

Skin interstitial fluid is found to be another source of biomarkers for early diagnosis, real-time monitoring, and disease control besides blood, urine, saliva, and sweat [64]. Biomarker detection via skin interstitial has shown numerous advantages [64]. Firstly, due to its simultaneous contact with tissues, cells, and capillaries, skin interstitial fluid not only contains a majority of blood biomarkers but also possesses a vast number of biomarkers that cannot be found in blood. Secondly, because skin interstitial fluid is close to the skin surface, the extraction of skin interstitial fluid is more accessible and less skin-damaging compared to that of blood. Thirdly, biomarkers from skin interstitial fluid reflect the local physiological states and can act as a precise indicator of local tissue events. Finally, it is easy to preserve and treat the extracted skin interstitial fluid, since no clotting will occur. Because of the minimal-invasive manner and the convenient application, microneedles have emerged as a novel method to detect a wide variety of biomarkers from skin interstitial fluid, including small molecule metabolites, nucleic acids, proteins, and even cells [65, 66]. A selective summary of biomarkers that can be detected by microneedles is given in Table 3. These detection microneedles mainly possess two working mechanisms. The first is absorbing the interstitial fluid with microneedles, extracting samples from microneedles, and analyzing the samples with other instruments [67–69]. The second is integrating all the detection processes into all-in-one microneedles by imparting them with the ability to specifically identify biomarkers [70].

### 4.1. Microneedles for Skin Interstitial Fluid Absorption

Generally, a microneedle array is connected with a reservoir to serve as a skin interstitial fluid absorption device [65, 67]. The microneedle array creates microchannels through the skin and forms a bridge between skin interstitial fluid and the reservoir, allowing the skin interstitial fluid to be absorbed by capillary forces or external pulling forces. The reservoir can be a microfluidic chip or a paper chip, collecting the extracted skin interstitial fluid and even providing the detection result. For example, microneedles were combined with a plasmonic paper on the supporting layer [67]. The plasmonic paper was coated with both recognition components for identifying the specific biomarker and augment components for conducting surface-enhanced Raman scattering (SERS). Once the skin interstitial fluid was absorbed through the microchannels and collected by the plasmonic paper, the SERS spectra of the targeted biomarker could be instantly acquired, giving quantified information.

**Figure 6:**
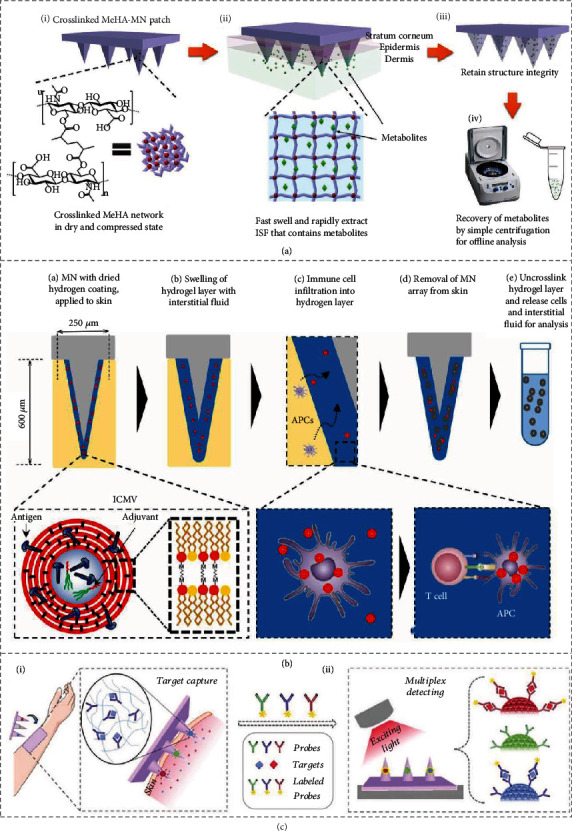
Biomarker detection via microneedles. (a) Swellable microneedles absorb skin interstitial fluid, and biomarkers are recovered by centrifugation. Reproduced with permission from Ref. [69], copyright 2017, Wiley-VCH. (b) Coating microneedles absorb skin interstitial fluid and detect specific immune cells. Reproduced with permission from Ref. [3], copyright 2018, American Association for the Advancement of Science. (c) Encoded microneedles specifically capture biomarkers and are decoded by fluorescence signals. Reproduced with permission from Ref. [70], copyright 2019, Wiley-VCH.

In spite of the efficacy, the integration of microneedles and the reservoir brings trouble and inconvenience to the fabrication and application. To simplify the device, researchers have presented microneedles with a superior swelling capacity [68]. Such microneedles can rapidly swell to absorb enough quantity of skin interstitial fluid during insertion. The extracted biomarkers can then be easily recovered from the microneedles by centrifugation, elution, and other mild methods. Notably, it is important to choose proper materials to fabricate these swellable microneedles. In one study, biocompatible water-affinity methacrylated hyaluronic acid (MeHA) was used to fabricate the microneedles (Figure 6(a)) [69]. It was found that the resultant microneedles swelled to approximately three times its original size and absorbed about 2.3 mg of skin interstitial fluid within 10 min. In addition, owing to the crosslinked network of MeHA, the microneedles could maintain their structural integrity and be completely removed from the skin.

In addition, Mandal et al. coated swellable alginate hydrogel on the surface of their microneedles, which could absorb both the skin interstitial fluid and cells to parallelly monitor immune responses, as schemed in Figure 6(b) [3]. The most distinctive feature of these microneedles was the encapsulation of molecular adjuvants and specific antigens within the swellable coating. Attributed to this, antigen-specific lymphocytes were recruited and enriched in the coating that absorbed the skin interstitial fluid and became swelling and porous upon insertion, enabling the further detailed analysis. By applying these microneedles to immunized mouse models and human skin samples, researchers demonstrated the practical functions of these microneedles in vaccination, immunotherapies, and other related biomedical fields. Due to all these features, it was anticipated that these microneedles could be integrated into microfluidic chips for cell separation, detection, and analysis.

### 4.2. All-in-One Microneedles

To realize specific biomarker detection and integration of all detection steps into the microneedles, probes that can specifically capture targeted biomarkers are coated on or encapsulated in microneedles [23, 70]. When these all-in-one microneedles are inserted into the skin, the probes will attract and enrich desired biomarkers quickly. After removal, biomarkers captured by the probes are then detected and analyzed by immunofluorescence, ELISA, etc., right on the microneedles. An example is shown in Figure 6(c), where antibody-decorated photonic crystal barcodes are placed inside the tips of microneedles [70]. After biomarker capture by the barcodes, fluorescently labelled antibodies were added to the encoded microneedles to form sandwich immunocomplexes with corresponding biomarkers. By differentiating the reflection peaks of the barcodes, the species of these biomarkers were distinguished; meanwhile, by reading the fluorescence intensity of the barcodes, the relative amounts of the biomarkers were obtained. As barcodes with different reflection peaks could be decorated by different probes, the encoded microneedles could realize multiplex detection and might be used as bioanalytical assays. With a similar principle, nucleic acid-capturing microneedles have also been generated. For example, using the peptide nucleic acid (PNA) as probes, Irvine et al. developed microneedles for detecting specific circulating nucleic acids [23]. Specifically, the alginate-PNA complex was coated on the surface of microneedles, enabling the microneedles to capture and enrich targeted circulating nucleic acids from the skin interstitial fluid. With the subsequent adding of a solution of DNA intercalator, the captured circulating nucleic acids could be directly visualized and imaged with a fluorescence scanner.

The combination of microneedles and electrochemical detection paves another path for the design of all-in-one microneedles. The electrode can be placed inside hollow microneedles and can reach the inner tissue with the assistance of microneedles [73]. Besides, the detecting agent such as hydrolase, which can react with the specific biomarker, is immobilized on the electrode. Then, the reaction product can be immediately detected by the electrode and transformed to an electrical signal. Based on the strength of the electrical signal, the concentration of the biomarker can be easily obtained. On the other hand, microneedles themselves can behave as the detecting electrode by mixing with conductive materials such as Pt nanoparticles and reduced graphene oxide [71]. In this case, a protective layer composed of PVP or other dissolvable polymers is coated on the microneedle electrode to avoid mechanical damage during microneedle penetration.

Cascade enzyme reactions could also be introduced into microneedle systems. Recently, Wang et al. designed a colorimetric microneedle patch for glucose level detection [72]. For this patch, calcium phosphate-encapsulated horseradish peroxidase (HRP) and 3,3′,5,5′-tetramethylbenzidine (TMB) were encapsulated in the supporting layer, while glucose oxidase (GOx) was encapsulated in the tip layer. Glucose in skin interstitial fluid would be converted into gluconic acid and H_2_O_2_ by GOx. The local acid environment then led to the breakdown of calcium phosphate and released HRP, which would react with TMB and produce visible color change in the presence of H_2_O_2_. Benefitting from the convenience and rapidity, this colorimetric patch was an ideal candidate for the portable, household glucose sensor.

## 5. Cargo Delivery

Oral administration, injection, and transdermal delivery are three main cargo delivery approaches [61, 74]. Oral administration is facile and convenient, while it is not suitable for infants and young children and the utilization rate is low due to the gastrointestinal absorption. As for injection, despite its efficiency, wide applicable population, and high utilization rate, the application process causes pain and requires the help of medical professionals. Although transdermal delivery can overcome the limitations of injection, traditional transdermal delivery means such as plaster, ointment, spray, etc., are still faced with the restriction of skin barrier, which brings about a low absorption rate and unsatisfactory delivery effects. Different from these traditional methods, microneedles, a novel and emerging transdermal delivery method, can open channels throughout the skin barrier without touching nerve endings or blood capillaries, thus achieving both effective delivery and good user experience [20]. Relying on the concentration gradient of cargos between microneedles and tissues, microneedles release their loaded cargos in a versatile, efficient, controllable, convenient, and safe manner [75]. Up to now, many different kinds of cargos [76] (e.g., small molecule drugs, proteins, and nanovehicles) have been delivered via microneedles (Table 4). Besides, these microneedles have been applied to different body sites [77, 78], including the skin, eyes, and vessels (Table 5).

### 5.1. Different Types of Cargos

#### 5.1.1. Small Molecules

Both water-soluble molecules and oil-soluble molecules can be encapsulated in microneedles by blending and copolymerizing with the microneedle tip materials. It is reported that microneedles can not only deliver antimicrobic drugs to treat infection-related diseases [79] but also release drugs with hormonal functions to regulate endocrinology and metabolism [80–82]. In addition, microneedles can carry anticancer drugs such as doxorubicin and docetaxel for tumor chemotherapy [83]. Notably, to enhance the therapeutic effects or eliminate the side effects, more than two kinds of small molecules are loaded in microneedles simultaneously. For example, dissolvable microneedles were developed for codelivery of a rapid antihypertensive drug-sodium nitroprusside and an antidote-sodium thiosulfate [84]. Such microneedles were demonstrated to achieve a rapid and potent reduction of blood pressure in rat models and effective suppression of side effects induced by sodium nitroprusside at the same time.

#### 5.1.2. Nucleic Acids

DNA vaccine can be delivered through microneedles to immune-cell-rich skin tissues, where microneedles protect the vaccine from degradation, facilitate a persistent vaccine stimulation, and induce an enhanced immune response [87]. To further elongate the immune time and amplify the immune response, DeMuth et al. stacked plasmid DNA, immune-stimulatory RNA, and biodegradable polycations layer by layer on the surface of microneedles [88]. The immune multilayer complex was implanted into the skin in response to the skin pH and existed in the skin from days to weeks. The kinetics was tailored by the composition of the complex. In addition to vaccination, microneedles can also deliver DNA for therapy [89, 90]. For instance, dissolvable microneedles were designed to release DNA aptamers that could block vascular endothelial growth factor (VEGF) and inhibit its function [91]. These microneedles were proved to be a promising therapeutic tool for the treatment of protein overexpression-related diseases.

#### 5.1.3. Protein or Peptides

A representative example of protein cargos delivered by microneedles is insulin. Compared to insulin injection, insulin delivery assisted by microneedles can avoid pain and repeated administration, while achieving a comparable hypoglycemic effect. Notably, to reduce the risk of hypoglycemia caused by insulin administration and realize self-regulation, the microneedles are made glucose-responsive, relying on chemical-reaction-induced disassembly of insulin complex [49, 92]. In addition to insulin, a variety of proteins and peptides including virus-related antigens [42], allergens [94], antibodies [95], collagen [96], tumor-related antigens [115], and some protein/peptide drugs [12, 116] can also be delivered by microneedles. For example, dissolvable microneedles that enabled enhanced cancer vaccination were reported [39]. Composed of an amphiphilic triblock copolymer, the microneedles generated nanomicelles, which linked to hydrophilic tumor antigens and encapsulated the hydrophobic immunologic adjuvant during their dissolution in the skin. Thus, the codelivery of both hydrophilic and hydrophobic components was achieved, and a significant level of humoral and cellular immunity was produced. Moreover, microneedles were proved to deliver calcitonin gene-related peptide (CGRP) antagonist peptide for pain control [15]. These microneedles were made from CMC and would dissolve to release their cargos upon penetration. The released CGRP antagonist peptide then competed with CGRP for CGRP receptors, blocked signals from sensory nerve endings, and finally inhibited neuropathic pain.

#### 5.1.4. Liquid Cargos

A large number of cargos are preserved in liquid form, which are required to be reformulated into solid products by extra processing steps and additives before loading in microneedles. However, the additional processes complicate the manufacturing, efficacy verification, and medical approval and may bring about activity loss of the cargos. Therefore, direct delivery of liquid cargos via microneedles is of necessity. There are mainly two strategies for liquid cargo delivery, the passive delivery and active delivery. The former is driven by diffusion action and can be realized by coating condensed liquid droplets on microneedle tips [117]. This passive delivery method can accurately control the amount of liquid cargos, while making it difficult to store these microneedles. The latter depends on applied high pressure such as pumping, ejection, and actuators and normally employs hollow microneedles [97]. This active delivery method is rapid and efficient but brings inconvenience to the application.

**Figure 7:**
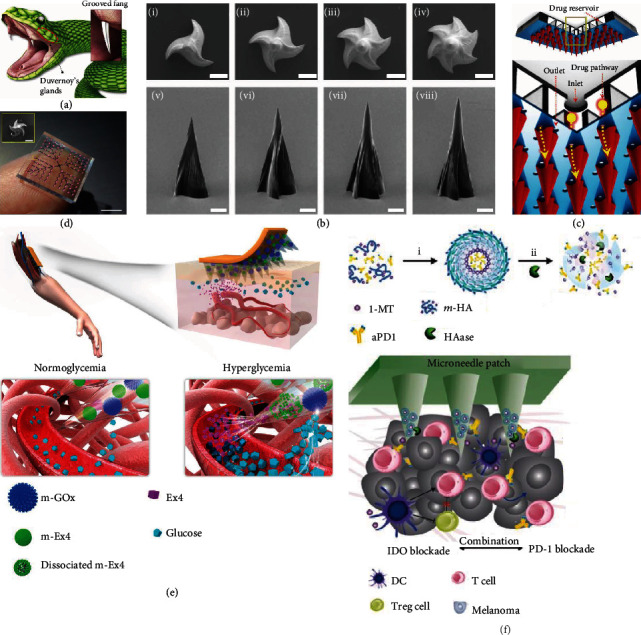
Microneedles for liquid cargo and nanovehicle delivery. (a–d) Fang-mimicked microneedles for liquid delivery: (a) schematic illustration of venom delivery by rear-fanged snakes; (b) SEM images of trigrooved (i, v), tetragrooved (ii, vi), pentagrooved (iii, vii), and hexagrooved (iv, viii) microneedles; (c) schematic illustration of microneedles bonded to a PDMS chamber; (d) corresponding optical photos. The scale bars are 100 *μ*m in (b) and the inset of (d) and 5 mm in (d). Reproduced with permission from Ref. [98], copyright 2019, American Association for the Advancement of Science. (e) Schematic illustrations of microneedles carrying m-GOx and m-EX4 for blood glucose control. Reproduced with permission from Ref. [105], copyright 2017, Springer Nature. (f) Schematic illustrations of microneedles carrying Apd1 and 1-MT in HA nanoparticles for tumor immunotherapy. Reproduced with permission from Ref. [118], copyright 2016, American Chemical Society.

Recently, novel snake-inspired microneedles for active liquid delivery independent of applied high pressure have been presented [98]. Attributed to the groove on their fangs, rear-fanged snakes could swiftly infuse venom into the prey's skin only based on surface tension and capillary action (Figure 7(a)). To emulate these fangs, grooves were made on the surface of microneedle tips. To note, more than one groove was generated to create multiple liquid flow paths and further improve delivery efficiency (Figure 7(b)). The fabricated microneedles were then bonded to a PDMS chamber as a liquid reservoir, and each microneedle tip was circled by a loop of microholes as liquid outlets (Figure 7(c) and (d)). When the microneedles were inserted into the skin, the penetrated part of the groove and the adjacent tissue formed a closed channel, while the upper part of the groove and the air formed an open channel. Under gentle thumb pressure, the liquid droplet passed through the open channel slowly but dramatically sped up as soon as it reached the closed channel due to the increased capillary force. It was found that such microneedles could successfully deliver the liquid formulations in vivo within 15 s, indicating their potential for simple and fast liquid cargo delivery.

#### 5.1.5. Nanovehicles

The delivery of insoluble or vulnerable cargos via microneedles has always been a challenge. As a satisfactory solution, these cargos can be first loaded in nanovehicles before they are placed into microneedles [99]. Specifically, the nanovehicle can be amphiphilic, encapsulating insoluble cargos in the core and increasing the solubility. Also, the nanovehicle can act as a shield, protecting the cargos from instant degradation and inactivation. Another function of nanovehicles carried by microneedles is the control of cargo release [100]. For example, concerning drugs with strong adverse effects, nanovehicles can extend the drug release time and reduce the amount of one-time release, which not only guarantee drug effectiveness but also avoid health risks. In addition, nanovehicles can elongate the retaining time of cargos in tissue, thus promoting the effects of the cargos and enhancing corresponding responses, such as the enhancement of immune response [101].

The integration of nanovehicles also imparts microneedle-guided transdermal delivery with responsiveness [102–104]. For example, Chen et al. immobilized GOx in copper phosphate particles (m-GOx), encapsulated exendin-4 (Ex4) in calcium phosphate particles (m-EX4), and loaded them together in microneedles (Figure 7(e)) [105]. In a hyperglycemic state, glucose reacted with GOx to induce a significant pH decrease, which did not impair the stability of m-GOx but triggered the dissociation of m-EX4, leading to a glucose-responsive EX4 release. Besides, responsive delivery of antitumor drugs could also be achieved. Gu et al. developed a microneedle system for tumor-microenvironment-responsive codelivery of two synergistic checkpoint inhibitors, anti-PD1 antibody (Apd1) and 1-methyl-DL-tryptophan (1-MT) [118]. 1-MT was covalently integrated with HA nanoparticles, and Apd1 was encapsulated in the core. When these nanoparticles were transported toward tumor sites by microneedles, hyaluronidase, an overexpressed enzyme in the tumor microenvironment, would dissolve HA and release the carried checkpoint inhibitors, followed by enhanced antitumor immune responses and reduced immunosuppression (Figure 7(f)).

#### 5.1.6. Cells or Cell Secretions

Microneedles can also serve as a platform for cell loading, which can either directly deliver cells into the skin or achieve transdermal delivery of active ingredients secreted by the loaded cells [10]. Previous work has accomplished the transportation of melanocytes into the skin via hollow microneedles for painless and effective leukoderma therapy [106], whereas there are still some issues that require future attention. Firstly, although the microneedles can inject the loaded cells into the dermis, the ability of these cells to function as normal remains unguaranteed. Secondly, the amount of the cells should be carefully determined and the cell activity during delivery should be evaluated. Eventually, this microneedle device should be further adjusted to deliver diverse kinds of cells, such as keratinocytes, fibroblasts, stem cells, and immunocytes.

**Figure 8:**
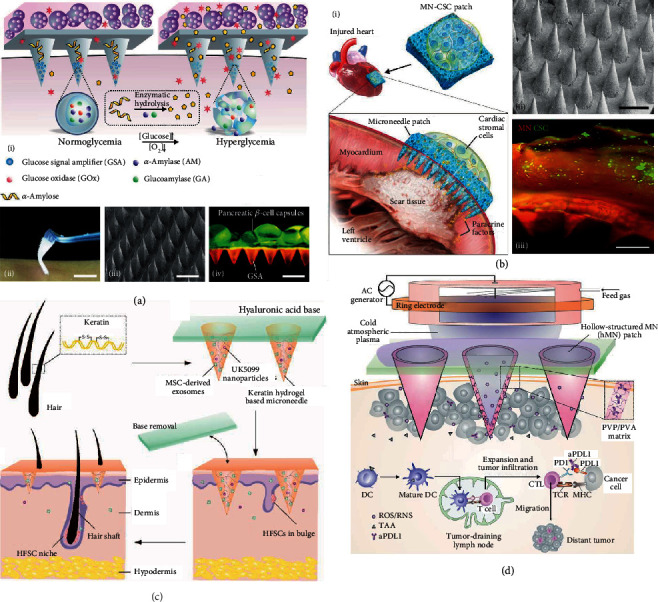
Microneedles for cargo delivery. (a) Microneedles carrying pancreatic *β*-cell capsules in the supporting layer for insulin delivery: (i) schematic illustrations of insulin release in response to hyperglycemia; (ii–iv) digital image (ii), SEM image (iii), and fluorescence microscopy image (iv) of the microneedle patch. Pancreatic *β*-cell capsules are stained with calcium AM. The scale bars are 1 cm in (ii) and 500 *μ*m in (iii, iv). Reproduced with permission from Ref. [107], copyright 2016, Wiley-VCH. (b) Microneedles bearing CSC-containing fibrin gel for heart regenerative factor delivery: (i) schematic illustrations of the microneedles applied to injured heart; (ii, iii) SEM image (ii) and fluorescence microscopy image (iii) of the microneedle patch. CSCs are labelled with DiO. Both scale bars are 500 *μ*m. Reproduced with permission from Ref. [108], copyright 2018, American Association for the Advancement of Science. (c) Schematic illustrations of keratin microneedles carrying exosomes and drugs for hair loss therapy. Reproduced with permission from Ref. [110], copyright 2019, American Chemical Society. (d) Schematic illustrations of hollow microneedles delivering CAP and ICI for cancer therapy. Reproduced with permission from Ref. [111], copyright 2020, PNAS.

Live cells can be cultured in the specially designed supporting layer of microneedles. The supporting layer provides adequate nutrients and necessary protection for the cells, and the functional cell excretions can be released through the microneedle tips. For example, Gu et al. presented live insulin-delivery microneedles by positioning pancreatic *β*-cell capsules in a microneedle supporting layer composed of crosslinked gel and cell media [107]. The loaded *β*-cells could receive amplified signals of elevated environmental glucose from the microneedle tips and smartly release insulin for glucose control (Figure 8(a)). The same group also provided cardiac microneedles by attaching cardiac stromal cell- (CSC-) containing fibrin gel to polymeric microneedles [108]. Such microneedles could be applied to the impaired heart and released CSC-secreted regenerative factors through the epicardium for therapeutic heart regeneration (Figure 8(b)). These cell-loading microneedles inspired the applications of microneedles in cell culture, where microneedles could protect cells, provide nutrients, keep cells alive, and enhance cellular communication both in vivo and in vitro.

#### 5.1.7. Others

Microneedles can release inactivated microbes like inactivated influenza virus vaccine for transdermal vaccination [1, 2], deliver cell extracts like exosomes for disease treatment [109, 110], and even assist the transport of cold atmospheric plasma (CAP) for cancer therapy [111]. For example, Yang et al. loaded mesenchymal stem cell-derived exosomes together with a small molecular drug inside separable microneedles constituted by hair-derived keratin (Figure 8(c)) [110]. Such microneedles were demonstrated to activate hair follicle stem cells by releasing their cargos, thus promoting hair follicle regrowth and providing a promising path for hair loss therapy. Besides, CAP-mediated immune checkpoint blockade therapy could be achieved by using hollow microneedles to successively release CAP and immune checkpoint inhibitors (ICI) into the tumor area (Figure 8(d)) [111]. It was found that compared with the single delivery of ICI, the combined delivery of both CAP and ICI induced an augmented antitumor immunity and an improved tumor killing rate.

### 5.2. Different Application Sites

As the largest organ of the human body, the skin is the major site where microneedles are applied. The carried cargos can pass through the microchannels created by microneedles, enter the inner tissues, diffuse via skin interstitial fluid, and finally reach where they function [98]. Since the concentration of cargos is the highest near the microneedles, skin administration works the best for dealing with near-skin diseases or vaccination, such as melanoma treatment and psoriasis treatment. For the prevention and treatment of some other diseases, microneedles intended for specific body sites including intestine [93], blood vessels [25, 85], ocular tissues [86], oral mucosa [112], genital tracts [114], and hearts [108] are also developed [78].

#### 5.2.1. Intestine

**Figure 9:**
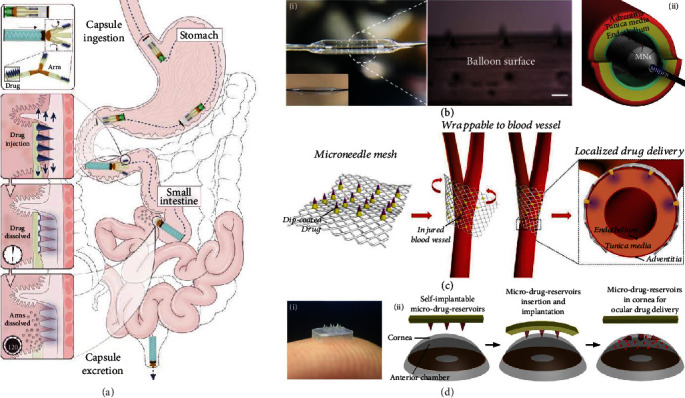
Microneedles applied to diverse body parts. (a) Schematic illustration of the oral insulin delivery process with intestine-applied microneedles. Reproduced with permission from Ref. [93], copyright 2019, Springer Nature. (b) Microneedles integrated on a drug-eluting balloon for endovascular drug delivery: (i) optical images of the microneedle balloon; (ii) schematic illustration of its application. The scale bar is 300 *μ*m. Reproduced with permission from Ref. [85], copyright 2020, Elsevier B.V. (c) Schematic illustrations of microneedles fixed to a surgical mesh and their application in perivascular drug delivery. Reproduced with permission from Ref. [25], copyright 2017, Elsevier B.V. (d) Separable microneedles for ocular drug delivery: (i) digital image of the microneedle patch; (ii) schematic illustration of its application. Reproduced with permission from Ref. [86], copyright 2018, Springer Nature.

Oral administration of protein drugs remains a challenge due to protein inactivation through the gastrointestinal tract. To realize the effective oral delivery, Abramson et al. designed a luminal unfolding microneedle injector (LUMI) device by integrating dissolvable microneedles with a three-armed injector and a compressed spring inside a capsule [93]. The protein drug, insulin, was encapsulated inside the microneedles. The capsule protected the embedded microneedles from the esophagus and stomach before it was digested in the small intestine (Figure 9(a)). The spring was then actuated, and the biodegradable arms of the injectors stretched to insert the microneedles into the tissue wall. Finally, the residues of the device were cleared away, with the microneedles and the arms dissolving and the nondegradable parts excreted from the anus. Importantly, LUMI with the chosen size and material was approved by the FDA, and the small intestine was reported to safely tolerate the microneedle penetration. Additionally, the monitoring of swine behavior for one week following the LUMI application showed that no discomfort occurred.

#### 5.2.2. Blood Vessel

High rates of vascular diseases such as intimal hyperplasia and neointimal formation have attracted increasing attention in vascular drug delivery. For this purpose, Lee et al. integrated microneedles with a drug-eluting balloon [85], as shown in Figure 9(b). When transported to the targeted vascular tissue, the balloon dilated, making the microneedles pierce through the endothelium and deliver the drugs into the tunica media. Through in vivo treatment for an atherosclerotic rabbit model, the delivery efficiency and the safety of the microneedle balloon were demonstrated. Also, microneedles could be suitable for perivascular drug delivery [25]. Such microneedles were fixed on a surgical mesh and were wrapped around the injured blood vessel during surgery (Figure 9(c)). The microneedle tips penetrated the adventitia and reached the tunica media to realize localized drug delivery.

#### 5.2.3. Ocular Tissue

Microneedles can break down ocular barriers and are applicable for efficient ocular drug delivery. For instance, Than et al. presented separable microneedles with crosslinked methacrylated HA as the microneedle tip material. During application, the supporting layer could be removed, leaving the drug-loaded, biodegradable, biocompatible tips in the cornea for sustained drug release (Figure 9(d)) [86]. Compared with conventional eye drops, microneedles can improve drug delivery efficiency and thus avoid repeated, high-dose instillation. In contrast to clinical intraocular injections, microneedles can ease the pain, lower the risks, and make possible the convenient household management of eye disorders.

#### 5.2.4. Others

As an immunologically rich site of the human body, oral mucosa is ideal for vaccination. Microneedles have been employed to assist oral mucosa vaccine delivery, which can carry various vaccine formulations, promote mucosa permeation, extend vaccine retention time, and improve immune effects [113]. Besides, to protect against sexually transmitted pathogens, microneedles for genital tract vaccine inoculation have also been developed. For example, vaccines were encapsulated in liposomes and then placed into dissolvable microneedles [114]. The microneedles could not only conquer the defending layer of mucus of the vagina but also overcome the tough barrier of densely lined epithelial cells. Thus, they could release the vaccines to inner tissue and elicit a robust immune response. Additionally, microneedles can function as heart patches. For instance, to treat acute myocardial infarction, microneedles integrated with CSCs have been developed to deliver necessary regenerative factors through cardiac muscles [108].

## 6. Conclusion and Perspective

The past few decades have seen the great leaps and bounds in microneedle fields. Much progress has been made including the popularization of biocompatible hydrogel microneedles, the improvement of microneedle fabrication strategies, and the exploration in clinical applications of microneedles. In the meanwhile, some reviews summarizing the classification of microneedles, their fabrication techniques, and applications in detection and drug delivery have been presented [119–121]. In recent years, microneedles have been imparted with novel and intriguing features such as skin adhesion, rapid dissolvability, separability, responsive ability, antibacteria, and explosive capacity to meet the complex requirements in actual use. These breakthroughs help microneedles find a wide range of applications such as detection of skin interstitial fluid biomarkers, early diagnosis, real-time monitoring, household drug administration, vaccination, and disease treatment. Herein, a general review of the emerging microneedles with smart properties and real-life applications is provided. Starting from the fabrication strategies, this review focuses on the advanced properties and accompanying functions of the microneedles. The practical values of these smart microneedles in forefront areas including biomedical engineering, clinical medicine, and biological science are also demonstrated. Different from other reviews, this review pays special attention to the unique properties of microneedles and the most recent advances in improving microneedles. Besides, this review systematically summarizes microneedle-based detection according to different detection principles for the first time. For the cargo delivery part, this review discusses both different types of cargos and diverse application sites, which is also a distinguishing feature from the existing reviews.

There is still a spacious room for the development in microneedle researches. To begin with, new materials can be employed to fabricate microneedles and the processing methods can be further improved. These materials should have sufficient mechanical strengths and skin adhesion ability, and the degradation products should be harmless. Naturally derived materials are also ideal choices. Besides, endowing microneedles with novel properties to adapt to more complicated functional requirements is another research direction. For example, breathable microneedles can improve the skin comfortability and multiresponsive microneedles can realize intelligently regulated drug delivery. Additionally, microneedles can perform their capabilities in many other biomedical fields, such as wound-repairing patches and 3D cell culture chips. Moreover, the comparison between microneedles and common clinical technologies is often neglected. Specifically, biomarker detection results acquired by microneedles should be further compared with the ones from conventional approaches to evaluate the accuracy and effectiveness of the microneedle-based detections. Also, it is anticipated to test the microneedles on large animals like pigs and monkeys, or even human patients. Furthermore, it is of great interest to integrate both diagnosis and treatment into microneedles. Under this circumstance, some microneedle tips act as biosensing modules to continuously monitor the biomarkers, while others serve as responsive drug delivery modules. Finally, microneedles have potential in paving a new path for wearable devices. For this purpose, the adhesion ability of microneedles should be further enhanced and the combination of microneedles and other platforms (e.g., triboelectric generators, microfluidics, and flexible electronics) is desired.

Despite the thriving scientific achievements on microneedles, there remains a large gap from academic researches to the practical products. This is manifested in that the types of microneedle products are limited with relatively simple features. Taking cosmetic microneedles as an example, smart microneedles consisting of responsive networks or containing responsive nanoparticles have been presented for acne vulgaris or obesity treatment at the laboratory level [47, 56, 122], whereas cosmetic microneedles on the market are just silicon and metal microneedles without carrying drugs, or common dissolvable microneedles without responsiveness. The challenges for clinical translation are listed as follows:

1. Inadequate proof of the clinical effectiveness. Microneedles have been widely proved effective in cell experiments and animal tests, but their validity in treating patients remain seldomly tested
2. Insufficient understanding of clinical needs. The gap between experimental assumptions and clinical needs is a critical obstacle faced by many emerging technologies including microneedles. It greatly hinders the further development of microneedles in clinical applications
3. Problem in using in resource-poor settings. How to preserve, transport, and dispose microneedle devices and how to train unexperienced medical staffs to use microneedles should be considered before microneedles are generalized to resource-poor areas
4. Lack of uniform standards. As an emerging medical device, microneedle devices still lack unified production and implementation standards, and the approval process is slow and long
5. Difficulty in large-scale manufacture. Although the fabrication of microneedles with simple structures has been simplified and popularized, the fabrication of multicomponent or specially structured microneedles is still time-consuming and technically dependent and requires delicate operations. These bring difficulties in manufacturing the smart microneedles on a large scale
6. Nonnegligible cost problem. Due to the abovementioned complicated fabrication techniques, storage, and approval process, the clinical use of microneedles can be relatively expensive

Thus, to move from status quo to FDA approval and clinical application scenarios, the repeatability and effectiveness of microneedle devices should be fully demonstrated through cell experiments, animal tests, and clinical trials. Besides, an in-depth understanding of human physiological environments, a deep investigation of the clinical demands, and portability and convenience of the microneedle systems can facilitate such clinical translation. Finally, it is of importance to establish uniform standards and baselines for microneedle devices, following endeavors in improving the fabrication process and suppressing costs.

## 7. Methodology

During the paper searching process, the main search engine used was Web of Science. Besides, some recently accepted papers were directly searched and downloaded from the online library of specific periodicals, such as ACS, RSC, Wiley, Nature, and Science. The main keyword was “microneedle”, and other keywords that were used at the same time included “fabrication”, “adhesion”, “separable”, “dissolvable”, “detection”, “delivery”, “liquid”, “cell”, “intestine”, and “cornea”. The initial number of papers accumulated and selected for review were three hundred and three. The representative, groundbreaking, original, or recent work was picked out, and the final number was one hundred and twenty-two.

## Figures and Tables

**Table 1:** Fabrication strategies for microneedles and their potential applications.

Strategy	Example	Advantage	Limitation	Application
Mold-based fabrication	Typical four-step method [15, 19–22]	Accurate, reusable, able to generate multicomponent microneedles, suitable for massive production, easy to operate	Inflexible, nonuniversal, unavoidable drug loss, confined by the molds, including time-consuming and technology-dependent steps	The most commonly used strategy, especially for massive production or industrial production
	Modification via surface coating [3, 23]	Simple, controllable, accurate, protective of bioactive cargos, suitable for massive production, easy to operate	Inflexible, nonuniversal, confined by the molds, including time-consuming and technology-dependent steps	A strategy based on a typical four-step method, applicable to carrying bioactive cargos
	Mold filling by imprinting [24, 25]	Accurate, protective of bioactive cargos	Inflexible, nonuniversal, unavoidable drug loss, confined by the molds, including time-consuming and technology-dependent steps, relying on extra equipment	A strategy based on a typical four-step method, applicable to carrying bioactive cargos
	Mold filling by spin coating [26]	Accurate, protective of bioactive cargos	Inflexible, nonuniversal, confined by the molds, including time-consuming and technology-dependent steps, relying on extra equipment	A strategy based on a typical four-step method, applicable to carrying bioactive cargos
	Mold filling by infiltrating or spraying [27]	Accurate, protective of bioactive cargos	Inflexible, nonuniversal, confined by the molds, including time-consuming and technology-dependent steps, relying on extra equipment	A strategy based on a typical four-step method, applicable to carrying bioactive cargos

Mold-free fabrication	Drawing lithography [16, 17]	Versatile, adjustable, economical, efficient, less-harmful, unrestricted by the molds, with simplified processes, with neglectable drug loss	Lacking accuracy, technology-dependent, only able to fabricate unilayer, cone-shaped microneedles	Applicable to carrying bioactive cargos
	Droplet-born air blowing [18]	Versatile, adjustable, mild, less-harmful, unrestricted by the molds, with simplified processes, with neglectable drug loss	Lacking accuracy, technology-dependent, only able to fabricate unilayer, cone-shaped microneedles	Applicable to carrying bioactive cargos
	Centrifugal lithography [28]	Versatile, adjustable, economical, mild, less-harmful, unrestricted by the molds, with simplified processes	Lacking accuracy, only able to fabricate unilayer, cone-shaped microneedles	Applicable to carrying bioactive cargos
	3D printing [29]	Accurate, unrestricted by the molds, suitable for elaborate synthesis, able to fabricate complicated patterns	Dependent on precision instrument, expensive, time-consuming	Suitable for fabricating microneedles with complicated structures
	Microlens-integrated technique [30]	Inexpensive, mold-free, flexible, adjustable, scalable, automatic	Involving harmful steps, fastidious about microneedle materials	Potential in massive production or industrial production

**Table 2:** Tip materials for dissolvable microneedles.

Material	Polymerization	Dissolving time	Ref.
Polyvinylpyrrolidone (PVP)	Photopolymerization or drying	~5 min	[2]
Hyaluronic acid (HA)	Drying or centrifugal evaporation	2-30 min	[37]
Polyvinyl alcohol (PVA)	Drying	2-30 min	[38]
Poly(ethylene glycol) (PEG)	Drying	Less than 30 min	[39]
Dextran and sorbitol	Drying	~5 min	[40]
Gelatin	Drying	~15 min	[41]
Hydroxyethyl starch (HES)	Drying	~10 min	[42]
Carboxymethyl cellulose (CMC)	Drying	2-30 min	[43]
Trehalose	Drying, dehydration	3-20 min	[44]
Poly-*γ*-glutamic acid (*γ*-PGA)	Drying	Less than 4 min	[45]

**Table 3:** Biomarkers detected by microneedles.

Biomarker	Example	Microneedle type	Ref.
Small molecule	Glucose, cholesterol, organophosphate, chloride, lactate, antibiotic, H_2_O_2_	Absorbing microneedles, all-in-one microneedles	[61, 69, 71, 72]
DNA	Pathogen DNA	Absorbing microneedles, all-in-one microneedles	[55]
RNA	miRNA	Absorbing microneedles, all-in-one microneedles	[23]
Protein	TNF-*α*, IL-1*α*, IL-1*β*, IL-6, IgG, virus protein, tyrosinase	Absorbing microneedles, all-in-one microneedles	[70, 73, 74]
Cell	Immune cells	Absorbing microneedles	[3]

**Table 4:** Cargos delivered by microneedles.

Type	Example	Function	Ref.
Small molecule	Kanamycin	Antibacteria	[79]
	Metformin	Diabetes treatment	[80]
	Triamcinolone acetonide	Hypertrophic scar therapy	[81]
	Finasteride	Androgenetic alopecia treatment	[82]
	Doxorubicin	Anticancer	[83]
	Sodium nitroprusside	Antihypertensive treatment	[84]
	Antiproliferative drugs	Vascular disease treatment	[25, 85]
	Besifloxacin, diclofenac	Eye disease treatment	[86]

Nucleic acid	DNA vaccine	Vaccination, cancer immunotherapy	[87–89]
	Short interfering RNA	Gene expression inhibition	[90]
	DNA aptamer	Block protein	[91]

Protein or peptide	Insulin	Diabetes treatment	[49, 92, 93]
	Virus-related antigens	Vaccination	[42]
	Allergen	Allergen skin testing or immunotherapy	[94]
	Antibody	Disease treatment	[95]
	Collagen	Cosmetics and wound healing	[96]
	Tumor-related antigens	Cancer immunotherapy	[39]
	CGRP antagonist peptide	Pain release	[15]
	DC101	Eye disease treatment	[86]

Liquid	Protein drug solution, small molecule drug solution, vaccine solution	Disease treatment, vaccination	[97, 98]

Nanovehicle	Liposome, mesoporous silica nanoparticles, polymer vesicles, mineralized nanoparticles	Disease treatment, vaccination, anticancer	[99–105]

Cell	Melanocyte	Leukoderma therapy	[106]

Cell secretion	Insulin secreted by *β*-cells, regenerative factors secreted by CSCs	Disease treatment	[107, 108]

Inactivated microbe vaccine	Inactivated influenza virus vaccine	Vaccination	[1, 2]

Cell extract	Tumor lysate	Cancer immunotherapy	[109]
	Exosome	Hair loss therapy	[110]

CAP	CAP	Anticancer	[111]

**Table 5:** Application sites of microneedles.

Application site	Microneedle property	Function	Ref.
Skin	Stiffness, swellable ability or porosity, biocompatibility	Biomarker detection	[70]
	Optional: biomarker capture ability, specificity		
	Stiffness, suitable delivery speed	Drug delivery, vaccination	[2, 11, 53, 92]
	Optional: protective effect, adhesion, responsiveness, separability, dissolvability, antibacteria, controllability		

Intestine	Biocompatibility, protective effect, separability or adhesion or dissolvability, biodegradation, suitable delivery speed	Drug delivery	[93]
	Optional: responsiveness, controllability		

Blood vessels	Biocompatibility, protective effect, biodegradation, suitable delivery speed	Drug delivery	[25, 85]
	Optional: scaffold function, mesh function, responsiveness, controllability		

Ocular tissues	Biocompatibility, separability or transparency or dissolvability, biodegradation, suitable delivery speed	Drug delivery	[86]
	Optional: responsiveness, controllability		

Oral mucosa	Biocompatibility, protective effect, biodegradation, suitable delivery speed	Vaccination	[112, 113]
	Optional: separability, dissolvability, adhesion, responsiveness		

Genital tracts	Biocompatibility, protective effect, biodegradation, suitable delivery speed	Vaccination	[114]
	Optional: separability, dissolvability, adhesion, responsiveness, adjustability		

Hearts	Stiffness, biocompatibility, protective effect, biodegradation, suitable delivery speed	Drug delivery	[108]
	Optional: dissolvability, adhesion, responsiveness, adjustability		
